# Comparative analysis of plasma metabolomics markers in patients with major depressive disorder and healthy controls

**DOI:** 10.1192/j.eurpsy.2022.835

**Published:** 2022-09-01

**Authors:** C. Homorogan, D. Nitusca, V. Enatescu, C. Moraru, C. Socaciu, C. Marian

**Affiliations:** 1 Victor Babes University of Medicine and Pharmacy, Biochemistry, Timisoara, Romania; 2 Victor Babes University of Medicine and Pharmacy Timisoara-Discipline of Psychiatry, Timisoara, Romania and Eduard Pamfil Psychiatry Clinic, Timisoara County Hospital, Psychiatry, Timisoara, Romania; 3 RTD Center of Applied Biotechnology BIODIATECH, SC Proplanta, Biotechnology, Cluj-Napoca , Romania

**Keywords:** metabolomics, Depression

## Abstract

**Introduction:**

Mood disorders, including depression, are diseases associated with an increased risk of several metabolic alterations. Metabolomics studies have proved their potential for detecting novel biomarkers of psychiatric diseases.

**Objectives:**

To analyze the plasma metabolite profiling of patients with major depressive disorder (MDD) compared to healthy controls.

**Methods:**

The blood samples were collected from 11 patients diagnosed with MDD and 11 healthy controls, and plasma was separated by centrifugation. The profiles of the metabolites in the plasma samples were determined by Ultra-High Performance Liquid Chromatography-Quadrupole Time of Flight Electrospray Mass Spectrometry (UHPLC-QTOF-MS) in positive mode. The chromatograms were processed by Compass DataAnalysis 4.2 using the Find Molecular Feature (FMF) method and Profile Analysis 2.1 (Bruker, Daltonics) was further used for matrix generation. The MetaboAnalyst online software was used for univariate and multivariate analysis. The mass/charge ratio (m/z values) determined by biostatistics were identified from the Lipidomic Gateway (www.lipidmaps.org) and Human Metabolomic Data Base (www.hmdb.ca).

**Results:**

We found 14 metabolites which could discriminate between cases and controls, having an area under the curve (AUC) in the receiver operating characteristic (ROC) analysis of higher than 0.6. Among these, only two metabolites passed the p<0.05 threshold of statistical significance, one being 2.5 more abundant (p<0.001) in the plasma of MDD patients compared to controls and the other being 1.7 more abundant (p=0.005) in MDD patients compared to controls.

**Conclusions:**

The only metabolite that passed the false discovery rate correction was putatively identified from the metabolomics database as being the phosphatidylcholine PC (16:0/16:0).

**Disclosure:**

No significant relationships.

